# PSMA-PET/CT Findings in Patients With High-Risk Biochemically Recurrent Prostate Cancer With No Metastatic Disease by Conventional Imaging

**DOI:** 10.1001/jamanetworkopen.2024.52971

**Published:** 2025-01-03

**Authors:** Adrien Holzgreve, Wesley R. Armstrong, Kevyn J. Clark, Matthias R. Benz, Clayton P. Smith, Loïc Djaileb, Andrei Gafita, Pan Thin, Nicholas G. Nickols, Amar U. Kishan, Matthew B. Rettig, Robert E. Reiter, Johannes Czernin, Jeremie Calais

**Affiliations:** 1 Ahmanson Translational Theranostics Division, Department of Molecular and Medical Pharmacology, David Geffen School of Medicine, University of California, Los Angeles; 2 Department of Nuclear Medicine, LMU University Hospital, LMU Munich, Munich, Germany; 3 ULCA-Caltech Medical Scientist Training Program, David Geffen School of Medicine, Los Angeles, California; 4 Department of Radiological Sciences, University of California, Los Angeles; 5 Department of Nuclear Medicine, University of Duisburg–Essen, Essen, Germany; 6 Jonsson Comprehensive Cancer Center, University of California, Los Angeles; 7 Department of Radiation Oncology, University of California, Los Angeles; 8 Department of Medicine, Veterans Affairs Greater Los Angeles Healthcare System, Los Angeles, California; 9 Department of Urology, University of California, Los Angeles

## Abstract

**Importance:**

The phase 3 randomized EMBARK trial evaluated enzalutamide with or without leuprolide in high-risk nonmetastatic hormone-sensitive prostate cancer. Eligibility relied on conventional imaging, which underdetects metastatic disease compared with prostate-specific membrane antigen–positron emission tomography (PSMA-PET).

**Objective:**

To describe the staging information obtained by PSMA-PET/computed tomography (PSMA-PET/CT) in a patient cohort eligible for the EMBARK trial.

**Design, Setting, and Participants:**

This post hoc, retrospective cross-sectional study included 182 patients from 4 prospective studies conducted from September 15, 2016, to September 27, 2021. All patients had recurrent prostate cancer after radical prostatectomy (RP), definitive radiotherapy (dRT), or salvage radiotherapy (SRT). Analysis was performed from January 2023 to July 2024.

**Exposures:**

Patients included had increasing prostate-specific antigen (PSA) levels greater than 1.0 ng/mL (after RP and SRT) or 2.0 ng/mL above the nadir value (after dRT), PSA doubling time of 9 months or less, and a serum testosterone level of 150 ng/dL or greater. Exclusion criteria were distant metastatic disease on radiographic imaging and prior hormonal or systemic therapy.

**Main Outcomes and Measures:**

Staging information obtained by PSMA-PET/CT in patients with nonmetastatic disease according to conventional imaging.

**Results:**

From 2002 patients screened, 182 (median age at PET/CT scan, 69 years [IQR, 64-73 years]) were included. Median prescan PSA levels were 2.4 ng/mL (IQR, 1.4-4.8 ng/mL) after RP (n = 91), 6.9 ng/mL (IQR, 3.5-18.5 ng/mL) after dRT (n = 39), 2.6 ng/mL (IQR, 1.6-5.2 ng/mL) after RP and SRT (n = 52), and 2.8 ng/mL (IQR, 1.7-6.6 ng/mL) overall (n = 182). Results of PSMA-PET were positive in 80% of patients (73 of 91) after RP, 92% of patients (36 of 39) after dRT, 85% of patients (44 of 52) after RP and SRT, and 84% of patients (153 of 182) overall. PSMA-PET detected any distant metastatic disease (miTxNxM1) in 34% of patients (31 of 91) after RP, 56% of patients (22 of 39) after dRT, 60% of patients (31 of 52) after RP and SRT, and 46% of patients (84 of 182) overall. Polymetastatic disease (≥5 lesions) was found in 19% of patients (17 of 91) after RP, 36% of patients (14 of 39) after dRT, 23% of patients (12 of 52) after RP and SRT, and 24% of patients (43 of 182) overall.

**Conclusions and Relevance:**

In a cohort of patients with high-risk hormone-sensitive prostate cancer without evidence of metastatic disease by conventional imaging, PSMA-PET results were positive in 84% of patients, detected M1 disease stage in 46% of patients, and found polymetastatic disease (≥5 lesions) in 24% of patients, suggesting that patients’ high-risk nonmetastatic hormone-sensitive prostate cancers are understaged by conventional imaging. The results challenge the interpretation of previous studies, such as the EMBARK trial, and support the evolving role of PSMA-PET for patient selection in clinical and trial interventions in prostate cancer. Further studies are needed to assess its independent prognostic value and use for treatment guidance.

## Key Points

**Question:**

What prostate-specific membrane antigen–positron emission tomographic/computed tomographic (PSMA-PET/CT) findings are present among patients with high-risk biochemically recurrent hormone-sensitive prostate cancer that is nonmetastatic as determined using conventional imaging?

**Findings:**

This cross-sectional post hoc analysis included 182 patients with high-risk nonmetastatic hormone-sensitive prostate cancer from 4 prospective studies who were eligible for the EMBARK study. Patients’ cancers were understaged by conventional imaging; PSMA-PET results were positive in 84% of patients, PSMA-PET detected M1 disease stage in 46% of patients and found polymetastatic disease (≥5 lesions) in 24% of patients.

**Meaning:**

Further studies are needed to assess the independent prognostic value of PSMA-PET and its use for treatment guidance.

## Teaser

This cross-sectional study describes the staging information obtained by prostate-specific membrane antigen–positron emission tomography/computed tomography (PSMA-PET/CT) among a patient cohort with high-risk biochemically recurrent prostate cancer with no metastatic disease found on conventional imaging.

## Introduction

Recurrent nonmetastatic hormone-sensitive prostate cancer (nmHSPC) is defined by increasing prostate-specific antigen (PSA) levels while naive or responsive to androgen deprivation therapy (ADT), and without evidence of metastasis on conventional imaging. In the setting of biochemical failure after definitive primary therapy, androgen receptor pathway inhibitors have shown clinical utility in both metastatic and chemotherapy-naive disease.^1,2,3^ The EMBARK (A Phase 3, Randomized, Efficacy and Safety Study of Enzalutamide Plus Leuprolide, Enzalutamide Monotherapy, and Placebo Plus Leuprolide in Men With High-Risk Nonmetastatic Prostate Cancer Progressing After Definitive Therapy) trial is a randomized, phase 3 study (NCT02319837) that evaluated the effect of enzalutamide plus ADT and enzalutamide monotherapy on patients with high-risk nmHSPC with increasing PSA concentrations after definitive therapy.^4^ Patients who received either enzalutamide plus ADT or enzalutamide monotherapy showed a significant increase in metastasis-free survival compared with those who received ADT plus placebo.^5^ Patients who qualified for the EMBARK trial were classified through negative results on conventional imaging, which underdetects metastatic disease in comparison with prostate-specific membrane antigen–positron emission tomographic (PSMA-PET) imaging.^6^ In this study, we aimed to describe the PSMA-PET findings in a cohort of patients from 4 prospective studies who met the inclusion criteria for the EMBARK trial.

## Methods

### Study Design, Setting, and Participants

This retrospective cross-sectional study was approved by the University of California, Los Angeles (UCLA) institutional review board and followed the principles outlined in the Declaration of Helsinki.^7^ We screened 2002 patients from 4 prospective study databases (NCT02940262, NCT03515577, NCT04050215, and NCT03582774) who were enrolled at UCLA from September 15, 2016, to September 27, 2021, to derive a cohort of patients with high-risk nmHSPC who underwent PSMA-PET imaging for increasing PSA levels. Patients provided oral and written informed consent to take part in these studies. Key inclusion criteria to reflect the EMBARK trial were increasing PSA level above 1.0 ng/mL (after radical prostatectomy [RP] and salvage radiotherapy [SRT]) or 2.0 ng/mL above the nadir value (after definitive radiotherapy [dRT]) (to convert to micrograms per liter, multiply by 1.0), PSA doubling time of 9 months or less, and serum testosterone level of 150 ng/dL or more (to convert to nanomoles per liter, multiply by 0.0347). Exclusion criteria similarly followed the EMBARK trial, defined as distant metastatic (M1) disease detected by any conventional imaging, prior hormonal neoadjuvant or adjuvant therapy at the time of dRT for more than 36 months, more than 6 months of short-course ADT with less than 9 months of washout before randomization, or advanced systemic therapy for prostate cancer. Clinical characteristics, including primary therapy, initial PSA, biopsy Gleason score (describing a composite of histopathologic patterns with 1 = small, uniform glands; 2 = more stroma between glands; 3 = distinctly infiltrative margins; 4 = irregular masses of neoplastic glands; and 5 = only occasional gland formation; the biopsy Gleason score is the sum of the most predominant and the worst patterns and ranges from 2 to 9, with 2 being associated with the best and 9 with the worst prognosis), RP pathologic findings, ADT history, most recent PSA levels, and PSA doubling times, were collected from electronic medical records and existing databases from the previously listed prospective clinical trials. This study followed the Strengthening the Reporting of Observational Studies in Epidemiology (STROBE) reporting guideline for cross-sectional studies.

### PSMA-PET/Computed Tomography Scan

All patients underwent ^68^Ga-PSMA-11 PET/computed tomography (CT) at UCLA. Imaging acquisition was performed as previously described.^8^ After injection of a median of 5.0 mCi of ^68^Ga-PSMA-11, PET images were recorded at a median uptake time of 61 minutes (IQR, 57-68 minutes). A total of 178 of 182 patients (98%) received a CT contrast agent. PSMA PET/CT images were interpreted in consensus by a board-certified nuclear medicine physician and a board-certified radiologist with access to all patient medical information. PSMA PET/CT findings (Prostate Cancer Molecular Imaging Standardized Evaluation [PROMISE] miTNM stage, lesion location, number of lesions) were collected from the clinical imaging reports and existing databases from the previously listed prospective clinical trials.

### Statistical Analysis

Statistical analysis was performed from January 2023 to July 2024. Patient baseline characteristics and scan findings are provided with summary statistics. Comparison of populations was used to evaluate the relative distribution (in percentages) of disease within groups. The Pearson χ^2^ test was used to assess the association between primary treatment groups and PSMA-PET–based staging categories as well as to compare the distributions in the patient cohort of this analysis with the original EMBARK study population. All statistical tests were 2-sided, and a threshold of *P* < .05 was considered to be statistically significant for rejection of the null hypothesis. Statistical analyses were performed using IBM SPSS Statistics, version 29 (IBM Corp).

## Results

### Patient Population

**Figure 1.**
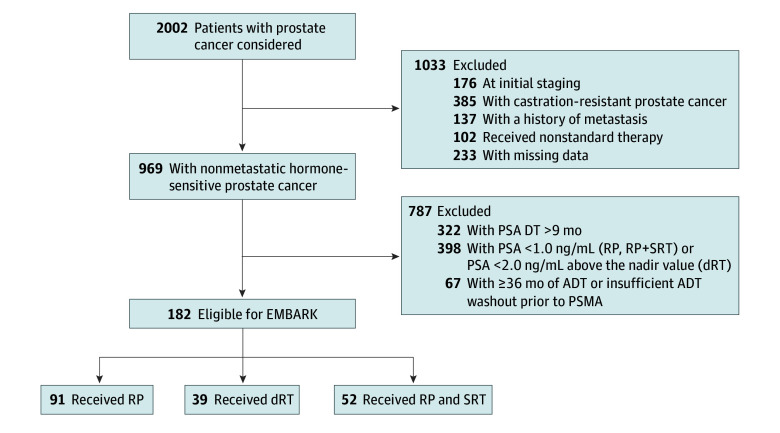
Study Flow Diagram ADT indicates androgen deprivation therapy; dRT, definitive radiotherapy; DT, doubling time; PSA, prostate-specific antigen; PSMA, prostate-specific membrane antigen; RP, radical prostatectomy; and SRT, salvage radiotherapy. To convert PSA to micrograms per liter, multiply by 1.0.

From the cohort of 2002 patients screened, 1033 were excluded: 176 at initial staging, 385 with castration-resistant disease, 137 with known metastatic disease, 102 who received nonstandard initial therapy (cryotherapy or high-intensity focused ultrasound), and 233 with missing data required for analysis. Of the remaining 969 patients, 322 had a PSA doubling time of more than 9 months, 398 had increasing PSA values less than 1.0 ng/mL (after RP and SRT) or less than 2 ng/mL above the nadir value (after dRT), and 67 had recent ADT or a washout period of 6 months or fewer; these patients were also excluded (Figure 1).

A total of 182 patients (median age at PET/CT scan, 69 years [IQR, 64-73 years]) with nmHSPC who met the EMBARK inclusion criteria were included in the analysis: 91 (50%) underwent RP, 39 (21%) received dRT, and 52 (29%) received SRT after RP.

The median time from primary therapy to PSMA-PET was 28 months (IQR, 8-62 months). The median time from primary therapy to biochemical recurrence (BCR) was 60 months (IQR, 19-250 months). Median prescan PSA levels were 2.4 ng/mL (IQR, 1.4-4.8 ng/mL) after RP, 6.9 ng/mL (IQR, 3.5-18.5 ng/mL) after dRT, 2.6 ng/mL (IQR, 1.6-5.2 ng/mL) after RP and SRT, and 2.8 ng/mL (IQR, 1.7-6.6 ng/mL) overall (Table 1). The median PSA doubling times were 3 months (IQR, 1.9-5.4 months) after RP, 3.3 months (IQR, 2.2-4.9 months) after dRT, 4 months (IQR, 2.6-5.5 months) after RP and SRT, and 3.6 months (IQR, 1.9-5.4 months) overall. The Gleason scores at diagnosis were 8 or higher for 35 of 91 patients (38%) after RP, 18 of 39 patients (46%) after dRT, 13 of 52 patients (25%) after RP and SRT, and 66 of 182 patients (36%) patients overall.

**Table 1.** Patient Characteristics

Characteristic	RP (n = 91)	dRT (n = 39)	SRT (n = 52)	Overall (N = 182)^a^
Age at PSMA-PET/CT, median (IQR), y	69 (64-72)	67 (63-77)	70 (66-73)	69 (64-73)
Last PSA value before enrollment, median (IQR), ng/mL	2.4 (1.4-4.8)	6.9 (3.5-18.5)	2.6 (1.6-5.2)	2.8 (1.7-6.6)
Time between last therapy and PSMA-PET/CT scan, median (IQR), mo	28 (8-62)	40 (26-72)	92 (53-124)	43 (18-93)
Initial PSA level, No. (%)				
<10 ng/mL	38 (41.8)	21 (53.8)	31 (59.6)	90 (49.5)
≥10 to <20 ng/mL	23 (25.3)	8 (20.5)	5 (9.6)	36 (19.8)
≥20 ng/mL	15 (16.5)	9 (23.1)	2 (3.8)	26 (14.3)
Unknown	15 (16.5)	1 (2.6)	14 (26.9)	30 (16.5)
Gleason score, No. (%)^b^				
≤6	1 (1.1)	5 (12.8)	3 (5.8)	9 (4.9)
7	55 (60.4)	15 (38.5)	34 (65.4)	104 (57.1)
≥8	35 (38.5)	18 (46.2)	13 (25)	66 (36.3)
Unknown	0	1 (2.6)	2 (3.8)	3 (1.6)
Primary tumor stage, No. (%)				
pT2	33 (36.3)	26 (66.7)	19 (36.5)	78 (42.9)
pT3a	30 (33)	2 (5.1)	23 (44.2)	55 (30.2)
pT3b	26 (28.6)	1 (2.6)	7 (13.5)	34 (18.7)
pT4	0	1 (2.6)	0	1 (0.5)
Unknown	2 (2.2)	9 (23.1)	3 (5.8)	14 (7.7)
Regional LN stage, No. (%)				
pN0	45 (49.5)	0	37 (71.2)	82 (45.1)
pN1	26 (28.6)	1 (2.6)	2 (3.8)	29 (15.9)
pNx	19 (20.9)	34 (87.2)	8 (15.4)	61 (33.5)
Unknown	1 (1.1)	4 (10.3)	5 (9.6)	10 (5.5)

Abbreviations: CT, computed tomography; dRT, definitive radiotherapy; DT, doubling time; LN, lymph node; PET, positron emission tomography; PSA, prostate-specific antigen; PSMA, prostate-specific membrane antigen; RP, radical prostatectomy; SRT, salvage radiotherapy.

SI conversion factor: To convert PSA to micrograms per liter, multiply by 1.0.

^a^ Included were 182 patients with high-risk nonmetastatic hormone-sensitive prostate cancer meeting the eligibility criteria for enrollment in the EMBARK trial at the time of their PSMA-PET/CT.

^b^ Describing a composite of histopathologic patterns with 1 = small, uniform glands; 2 = more stroma between glands; 3 = distinctly infiltrative margins; 4 = irregular masses of neoplastic glands; and 5 = only occasional gland formation; the biopsy Gleason score is the sum of the most predominant and the worst patterns and ranges from 2 to 9, with 2 being associated with the best and 9 with the worst prognosis.

Compared with the original EMBARK study cohort of 1068 patients at 244 sites,^5^ we included significantly fewer patients treated with combined RP and SRT (29% vs 49%; *P* < .001) and significantly more patients treated with RP alone (50% vs 25%; *P* < .001). Patients in our study had a lower median PSA doubling time compared with the EMBARK study population (3.6 vs 4.9 months) and a lower median serum PSA level at enrollment (2.8 vs 5.2 ng/mL; patient-individual PSA-related values of the EMBARK trial were not available for statistical comparison).

### PSMA-PET Findings

**Figure 2.**
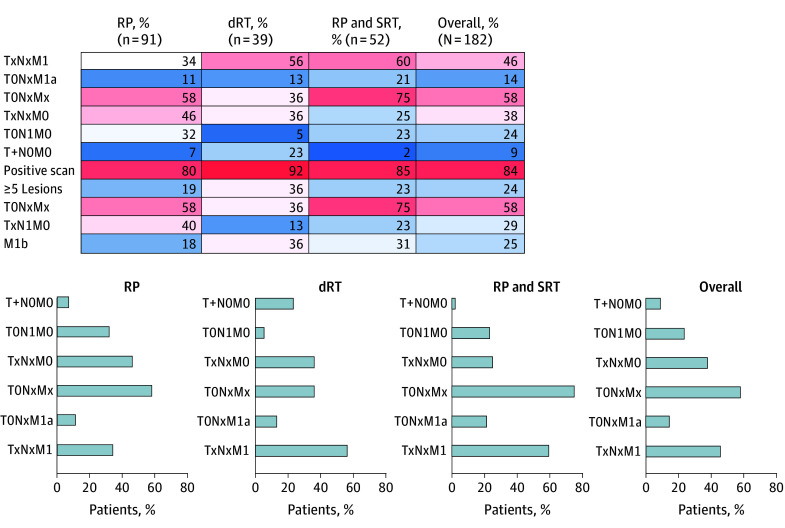
Disease Distribution by Primary Treatment dRT indicates definitive radiotherapy; RP, radical prostatectomy; and SRT, salvage radiotherapy.

Table 2 provides an overview of PSMA-PET results. The metastatic burden classification by PSMA-PET is shown in Table 3. The disease distribution by primary treatment as depicted by PSMA-PET is shown in Figure 2. PSMA-PET findings were positive in 80% of patients (73 of 91) after RP, 92% of patients (36 of 39) after dRT, 85% of patients (44 of 52) after RP and SRT, and 84% of patients (153 of 182) overall. PSMA-PET–detected disease was localized only to the prostate fossa (mi T+N0M0) in 7% of patients (6 of 91) after RP, 23% of patients (9 of 39) after dRT, 2% of patients (1 of 52) after RP and SRT, and 9% of patients (16 of 182) overall, and T+ status was significantly more frequent in the dRT group (23% [9 of 39] vs 7% in the RP group [6 of 91], 2% in the RP and SRT group [1 of 52], and 9% overall [16 of 182]; *P* < .001). PSMA-PET detected pelvic nodal disease (miTxN1M0) in 40% of patients (36 of 91) after RP, 13% of patients (5 of 39) after dRT, 23% of patients (12 of 52) after RP and SRT, and 29% of patients (53 of 182) overall. PSMA-PET detected any distant metastatic disease (miTxNxM1) in 34% of patients (31 of 91) after RP, 56% of patients (22 of 39) after dRT, 60% of patients (31 of 52) after RP and SRT, and 46% of patients (84 of 182) overall, and M1 status was significantly less frequent in the RP group (34% [31 of 91] vs 56% in the dRT group [22 of 39], 60% in the RP and SRT group [31 of 52], and 46% overall [84 of 182]; *P* = .005). PSMA-PET detected metastatic nodal only disease (N1 and/or M1a) in 44% of patients (40 of 91) after RP, 15% of patients (6 of 39) after dRT, 44% of patients (23 of 52) after RP and SRT, and 38% of patients (69 of 182) overall and osseous disease (M1b) in 18% of patients (16 of 91) after RP, 36% of patients (14 of 39) after dRT, 31% of patients (16 of 52) after RP and SRT, and 25% of patients (46 of 182) overall. Polymetastatic disease (≥5 lesions) was found in 19% of patients (17 of 91) after RP, 36% of patients (14 of 39) after dRT, 23% of patients (12 of 52) after RP and SRT, and 24% of patients (43 of 182) overall.

**Table 2.** PSMA-PET/CT Results

Site	PSMA-positive patients (N = 182), No. (%)	PSMA-positive lesions, No.	Mean No. of lesions in PSMA-positive cases
Overall	152 (83.5)	601	3.9
Prostate bed (T+)	46 (25.3)	49	1.1
Pelvic LN (N+)	101 (55.5)	270	2.6
Internal iliac	45 (24.7)	64	1.4
External Iliac	48 (26.4)	68	1.4
Common iliac	39 (21.4)	74	1.8
Obturator	11 (6.0)	14	1.3
Perirectal	5 (2.7)	9	1.8
Presacral	18 (9.9)	31	1.7
Other pelvis	4 (2.2)	10	2.5
Extrapelvic LN (M1a)	52 (28.6)	162	3.1
Inguinal	1 (0.5)	1	1.0
Retroperitoneal	45 (24.7)	105	2.3
Upper diaphragm	24 (13.2)	56	2.3
Bone (M1b)	46 (25.3)	106	2.3
Lung (M1c)	8 (4.4)	14	1.8

Abbreviations: CT, computed tomography; LN, lymph node; PET, positron emission tomography; PSMA, prostate-specific membrane antigen.

**Table 3.** Metastatic Burden Classification by PSMA-PET/CT

Disease burden categorization	Patients, No. (%)			
	RP (n = 91)	dRT (n = 39)	SRT (n = 52)	Overall (N = 182)
Nonmetastatic	60 (65.9)	17 (43.6)	21 (40.4)	98 (53.8)
Oligometastatic (1 lesion)	10 (11.0)	7 (17.9)	16 (30.8)	33 (18.1)
Oligometastatic (2-4 lesions)	17 (18.7)	10 (25.6)	11 (21.2)	38 (20.9)
Polymetastatic (>≥5 lesions)	4 (4.4)	5 (12.8)	4 (7.7)	13 (7.1)

Abbreviations: CT, computed tomography; dRT, definitive radiotherapy; PET, positron emission tomography; PSMA, prostate-specific membrane antigen; RP, radical prostatectomy; SRT, salvage radiotherapy.

## Discussion

In this study, we aimed to contextualize how PSMA-PET may influence the interpretation of the EMBARK trial results.^5^ In this retrospective study of 182 patients with nmHSPC eligible for the EMBARK trial (based on conventional imaging), we demonstrated that PSMA-PET detected metastatic disease in 46% of all patients, suggesting that a significant number of patients have disease that is understaged by conventional imaging.

The EMBARK trial demonstrated a significant survival benefit. Numerous studies have demonstrated the association of PSMA-PET findings with management decisions across multiple disease stages; however, there are limited data to support the benefit of management alterations.^9,10,11^ The ARCHES (A Multinational, Phase 3, Randomized, Double-blind, Placebo-controlled Efficacy and Safety Study of Enzalutamide Plus Androgen Deprivation Therapy [ADT] Versus Placebo Plus ADT in Patients With Metastatic Hormone Sensitive Prostate Cancer [mHSPC]) trial is a randomized phase 3 clinical trial of enzalutamide that reported outcomes similar to EMBARK in the metastatic hormone-sensitive prostate cancer setting.^12^ The trial stratified patients based on disease burden using conventional imaging and demonstrated the potential to use enzalutamide in the treatment of patients with low-volume disease.^12^ Additional consideration should be given to the eligibility of these patients for metastasis-directed therapy as an adjunct or predecessor to systemic androgen receptor pathway inhibitor therapy. The EMBARK trial selected patients meeting high-risk criteria by the European Association of Urology (EAU) BCR risk grouping, given a mandatory PSA doubling time of less than 9 months.^13^ The EAU BCR risk groupings were derived from patient cohorts evaluated using conventional imaging techniques.^14^ Integrating the EAU risk grouping with PSMA-PET, in 2023, Leplat et al^15^ reported significantly higher positivity rates in patients with high-risk BCR (59% vs 36%; *P* < .001), with 49% of cases with metastatic disease vs 31% in patients with low-risk BCR, all of which were oligometastatic in patients with low-risk BCR. In 2024, Scharl et al^16^ reported PSMA-guided SRT outcomes based on EAU BCR groupings, with a 3-year metastasis-free survival of 94.4% in patients with low-risk BCR vs 87.6% in patients with high-risk BCR (*P* = .005). Conversely, an analysis by Dong et al^17^ suggested that BCR risk groups define patients who benefit most from a PSMA-PET/CT scan in the case of BCR. In this context, a potential clinical benefit associated with PSMA-PET could be the identification of patients to be safely treated with local radiotherapy or stereotactic body radiotherapy, including a potential curative perspective in some cases. The implications of the EMBARK trial regarding the choice for metastasis-directed therapy or SRT vs enzalutamide in patients with increasing PSA levels are further discussed in a comment by Einstein et al,^18^ enlightening the approaches of metastasis-free survival vs treatment-free survival.

In addition, PSMA-PET/CT may lead to downstaging compared with conventional imaging. In a study including 167 patients across disease stages, bone scans were shown to have a high rate of false-positive results compared with PSMA-PET.^19^ Another retrospective study at 4 international sites comparing high-volume and low-volume disease as defined by the CHAARTED (ChemoHormonal Therapy Versus Androgen Ablation Randomized Trial for Extensive Disease in Prostate Cancer) criteria with PSMA-PET–based disease load similarly found that stage migration from conventional imaging to PSMA-PET occurs both by upstaging and by downstaging in patients with metastatic hormone-sensitive prostate cancer.^20,21^ The actual significance of such upstaging or stage migration on treatment outcomes and appropriate treatment management is difficult to assess and not yet known.

Prospective studies investigating the association between PSMA-defined risk groups and patient outcomes are warranted. PSMA-PET was highly associated with response to SRT and was associated with 3-year freedom from progression more accurately than clinical factors such as PSA level or Gleason score.^22^ In the ORIOLE (Observation vs Stereotactic Ablative Radiation for Oligometastatic Prostate Cancer) phase 2 randomized study, radiotherapy coverage of PSMA-positive disease decreased the risk of new lesions at 6 months (16% vs 63%; *P* = .006).^23^ In a large cohort of 1612 patients with prostate cancer including all disease stages, Karpinski et al^24^ reported that PSMA-PET standardized PROMISE criteria were accurate for estimation of overall survival, outperforming major established clinical risk tools. Efforts to prospectively analyze the benefit associated with PSMA-PET vs traditional imaging include an ongoing prospective multicenter study (Primary Staging of Prostate Cancer: A Randomized Controlled Trial Comparing 18F-PSMA-1007 PET/CT to Conventional Imaging [PRISMA-PET]; NCT05123300) that plans to include 448 patients, randomized 1:1 to either traditional imaging or PSMA-PET/CT. The study aim is to assess whether PSMA-PET/CT increases progression-free survival and quality of life.

### Limitations

The analysis performed in this study has limitations in its design and comparability with the EMBARK trial. Our analysis included significantly fewer patients undergoing SRT and more patients undergoing RP alone compared with the EMBARK trial. In our study, the SRT group most frequently showed metastatic disease according to results of PSMA-PET/CT: TxNxM1 in 60% of cases in the SRT group. Thus, our results may underestimate the actual proportion of patients with PSMA M1 disease in the EMBARK trial.

Furthermore, the lower median serum PSA level at enrollment translates to a lower disease burden in our cohort, indicating that our results may further underestimate the actual disease burden of patients included in the EMBARK trial, as PSMA-PET detection rates significantly increase in association with the PSA level.^25^ This was a cross-sectional analysis with no longitudinal follow-up. Further studies are needed to understand the association of PSMA-PET upstaging with clinical outcomes. The retrospective nature of this study precluded systematic baseline imaging as would be standard for true clinical trial enrollment.

Finally, even if PSMA-PET is the imaging modality with the best diagnostic accuracy for prostate cancer staging, it can lead to false-positive metastasis findings (positive predictive value in BCR, 0.84%^25^), especially in the bone. Here, we cannot estimate the rate of false-positive findings nor its association with patient outcomes.

## Conclusions

In this cross-sectional study of patients with high-risk nmHSPC and PSA doubling time of less than 9 months who were eligible for the EMBARK trial, PSMA-PET findings were positive in 84% of patients, detected M1 disease in 46% of patients, and found polymetastatic disease (≥5 lesions) in 24% of patients. PSMA-PET provides novel additional risk stratification for patients with high-risk nmHSPC without distant metastasis based on conventional imaging. Further studies are needed to assess its potential independent prognostic value and its use for treatment guidance. Integration of PSMA-PET in major industry-sponsored clinical trials for secondary end points analyses is warranted.

## Notes

Supplement.
Data Sharing Statement
