# Supplementary material for: PSMA-PET/CT Findings in Patients With High-Risk Biochemically Recurrent Prostate Cancer With No Metastatic Disease by Conventional Imaging
Source: JAMA Netw Open. 2025 Jan 3;8(1):e2452971. doi: 10.1001/jamanetworkopen.2024.52971 (PMC11699533; doi:10.1001/jamanetworkopen.2024.52971)
Supplement: Supplement. — Data Sharing Statement [file jamanetwopen-e2452971-s001.pdf]

Data Sharing Statement

Holzgreve. PSMA-PET/CT Findings in Patients With High-Risk Biochemically Recurrent Prostate Cancer With No Metastatic Disease by Conventional Imaging. *JAMA Netw Open*. Published January 03, 2025. doi:10.1001/jamanetworkopen.2024.52971

Data
Data available: No

Additional Information
Explanation for why data not available: The aggregated data generated in this study are available in this article. Further data are available upon reasonable request from the corresponding author.
